# More Severe Extratemporal Damages in Mesial Temporal Lobe Epilepsy With Hippocampal Sclerosis Than That With Other Lesions: A Multimodality MRI Study

**DOI:** 10.1097/MD.0000000000003020

**Published:** 2016-03-11

**Authors:** Wei Wei, Zhiqiang Zhang, Qiang Xu, Fang Yang, Kangjian Sun, Guangming Lu

**Affiliations:** From the Department of Medical Imaging (WW, ZZ, QX, GL), Department of Neurology (QX), Department of Neurosurgery (FY), Jinling Hospital, Nanjing University School of Medicine, and State Key Laboratory of Analytical Chemistry for Life Science (ZZ, GL), Nanjing University, Nanjing, China.

## Abstract

Mesial temporal lobe epilepsy with hippocampal sclerosis (mTLE-HS) presents different clinical presentations from that with other lesions (OL). It is significant to investigate the neural mechanism underlying the different clinical presentations using neuroimaging study.

Thirty mTLE patients with mTLE-HS, 30 mTLE patients with other lesions (mTLE-OL), and 30 age- and sex-matched healthy controls were involved. Amplitude of low-frequency fluctuation (ALFF) analysis-based resting-state functional magnetic resonance imaging (fMRI) and voxel-based morphometry (VBM) based morphometric MRI were employed to describing functional and structural imaging alterations in mTLE. Imaging parameters of ALFF and gray matter volume (GMV) were compared among groups and correlated with clinical variables and cognitive scores.

For parameter of ALFF, both patient groups of mTLE-HS and mTLE-OL showed decrease in the frontal cortices relative to the healthy controls; mTLE-HS showed more decrease in the prefrontal and brain default regions relative to mTLE-OL. For GMV, both patient groups showed decrease in the frontal cortex, thalamus, and cerebellum; mTLE-HS showed more GMV decrease relative to the mTLE-OL, also mainly in the prefrontal and brain default regions. In both patient groups, the prefrontal regions showed negative correlation between GMV and epilepsy duration.

This work revealed distinct alteration patterns of functional and structural brain organizations in mTLEs with different forms. MTLE-HS, despite with smaller lesion size of the pathological focus, presented more severe functional and structural damages in the extratemporal regions than mTLE-OL. The findings provided imaging evidence to support the proposal that mTLE-HS is a special epilepsy syndrome.

## Abstract 2

Supplemental Digital Content is available in the text

## INTRODUCTION

Mesial temporal lobe epilepsy (mTLE) is the most common type of human intractable epilepsy. The pathological substrates of mTLE could be either, most usually, hippocampal sclerosis (HS), or other foreign-tissue lesions in the mesial temporal structures, such as low-grade ganglioglioma, vascular malformation, etc.^1^ Specifically, mTLE associated with HS (mTLE-HS) is recognized as a well-defined epilepsy syndrome,^2^ shows different clinical characteristics from mTLE associated with other lesions (mTLE-OL). Relative to mTLE-OL, patient with mTLE-HS has earlier epilepsy onset,^3,4^ less secondary generalized seizures,^2,5^ higher incidence of ‘initial precipitating incidents (IPI),^6^ and more severe high-level cognitive impairments.^2,7,8^ Inconsistent evidence^9–11^ indicates that mTLE-HS may have more successful surgical outcome. It is of clinical and scientific significances to investigate the neural mechanisms underlying the different clinical presentations in these 2 types of mTLE.^8^ However, so far there is still lack of imaging study on this issue.

Multimodality MRI has been the most important neuroimaging tool to detect the functional and structural changes of human brain in epilepsy.^12^ Voxel-based morphometric (VBM) is a fully automated computerized quantitative analysis technique for estimating regional gray-matter alteration in epilepsy.^13^ Resting-state functional magnetic resonance imaging (fMRI) allows measuring baseline brain activity in physiological and pathological states, and amplitude of low-frequency fluctuation (ALFF) analysis can depict regional property of resting-state brain blood oxygenation level dependent fluctuations.^14^ ALFF-based resting-state fMRI has been used to measure brain activity corresponding to cognitive deficits and epileptic discharges in epilepsy.^15,16^ In the present study, we combined ALFF-based resting-state functional MRI^14^ and voxel-based morphometric (VBM)-based structural MRI^17^ techniques, to explore the differences of functional and structural alterations within the extratemporal regions between mTLEs with HS and OL. We hypothesized that in addition to difference of pathological features in the epileptogenic focus, there might be regional differences of brain structural and functional alterations in the extra-temporal regions, according to the concept of epilepsy network.^18^ The work was anticipated to provide imaging evidence supporting the proposal that mTLE with HS might be a special syndrome, and shed lights on the mechanism of brain organizations in mTLE with different causes.

## MATERIALS AND METHODS

### Participants

This study enrolled 70 patients with mTLE-HS and mTLE-OL. For patients with mTLE-OL, 30 cases with valid data were adopted from 35 consecutive patients with unilateral mTLE-OL from August 2011, to August 2014 in Jinling Hospital (3 cases with large head motion for functional data, one failure of functional data acquisition and the other with failure of structural data acquisition in MRI). Thirty patients were finally adopted. In addition, 30 patients with unilateral mTLE-HS were randomly selected from data pool of our research group. The lateralization of epileptogenic focus, age and genders of mTLE-HS matched with those of mTLE-OL. Data were selected from 115 consecutive cases from 2009 to 2014. Diagnosis of mTLE was based on International League against Epilepsy classification 2006: Typical symptoms of mTLE, including complex partial seizures. Electroencephalogram (EEG) findings. Predominantly left/right-sided frontotemporal/temporal lobes epileptic discharges in the left/right mTLE patients shown by interictal and ictal scalp EEGs. Specifically, for mTLE-OL, all cases had identifiable structural MRI abnormalities in unilateral (14 left- and 16 right-sided) temporal lobe (cavernous hemangioma: n = 10, arachnoid cyst: n = 6, gliosis: n = 6, low-grade glioma and dysembryoplastic neuroepithelial tumor: n = 5, cortical dysplasia: n = 2). Eighteen patients were confirmed by postoperative histopathology, and 12 were identified by MRI. For mTLE-HS, HS was diagnosed by clinical MRI presentations, including decreased hippocampal volume,^19^ high signal intensity on T2WI and abnormal MR spectroscopy signal (NAA/Cho + Cr < 0.6).^20^ Thirty cases were confirmed by pathological outcome. Moreover, other clinical information including history of IPI, history of secondary generalized seizures, epilepsy duration, response for antiepileptic medication, and surgical outcomes is summarized in Table 1.

**TABLE 1:**
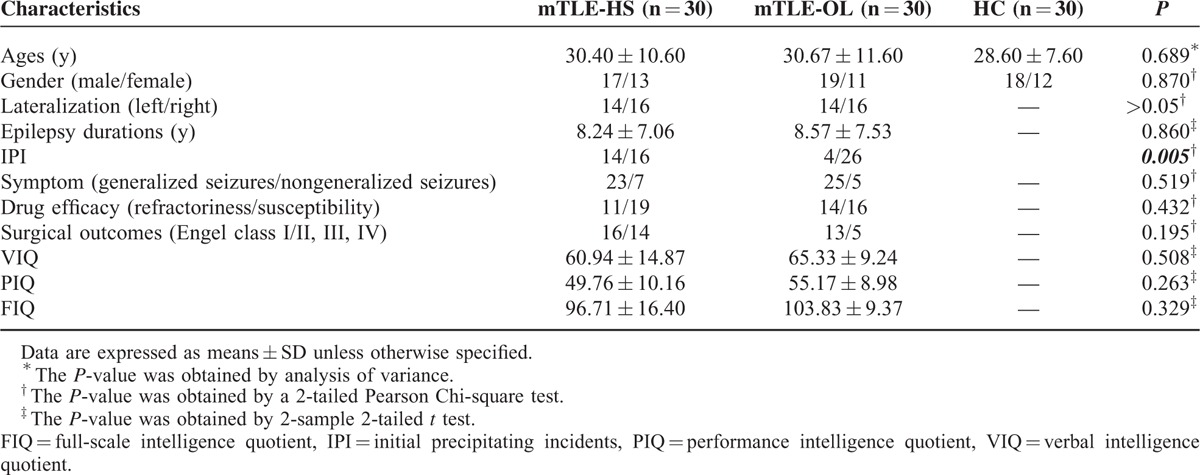
Clinical Data of the mTLE Patients and HC

All patients had taken antiepileptic drug medication (valproate: n = 26; carbamazepine: n = 22; lamotrigine: n = 11; phenytoin: n = 9; traditional Chinese medicine: n = 7; topiramate: n = 6; oxcarbazepine: n = 6; phenobarbital: n = 3), 25 patients were drug refractory and had taken more than 2 antiepileptic drug. Forty-eight patients underwent surgical treatment of anterior temporal lobectomy. Two clinical variables, epilepsy duration and estimated number of lifetime seizures were used for describing the progression of epilepsy. Epilepsy duration was defined as time span from the onset time of habitual seizures to the scan time. Seventeen patients with mTLE-HS and 11 patients with mTLE-OL had taken neuropsychological tests using the Wechsler Intelligence Scale third edition (WMS III) to evaluate the cognitive function of the patients (Table 1).

Moreover, 30 age- and gender-matched healthy controls (HC) were enrolled in this study. None of them had history of psychiatric or neurological disorders. This research was approved by the Medical Ethics Committee in Jinling Hospital, Nanjing University School of Medicine, and written informed consent was obtained from each participant.

### Data Acquisition

All patients and healthy controls underwent structural and functional data scanning on a Siemens Trio 3T MRI scanner in Jinling Hospital, Nanjing. Subjects were instructed to be eyes closed and not to think of anything in particular, a foam padding was used to minimize head movement. Functional images were acquired using a single-shot, gradient-recalled echo planar imaging sequence (repetition time = 2000 milliseconds, echo time = 30 milliseconds, field of view = 240 × 240 mm^2^, in-plane matrix = 64 × 64 and flip angle = 90°). A total scan time for each subject was 500 seconds, and 30 transverse slices (slice thickness = 4 mm, interslice gap = 0.4 mm) were acquired aligning along the anterior–posterior commissure line.

Structural images were acquired using a magnetization-prepared rapid gradient-echo sequence (repetition time = 2300 milliseconds, echo time = 2.98 milliseconds, flip angle = 9°, field of view = 256 × 256 mm^2^, and slice thickness = 1 mm). One hundred seventy-six slices of high-resolution 3D T1-weighted anatomical images were accessed in the sagittal orientation covering the whole brain. Moreover, the other sequences for clinical diagnosis, including T1WI, T2WI, DWI, and T2-FLAIR, were also acquired, and did not be presented here.

### Data Processing

Image preprocessing was carried out using Matlab-based toolkit of SPM8 (www.fil.ion.ucl.ac.uk/spm). Firstly, all functional and structural images of the right mTLE patients were left-to-right flipped, in order to enlarge the sample size and allow analysis of all patients as a homogeneous group.

#### Processing of Structural Imaging Data

Considering the possible effect of lesion on spatial normalization, we used a cost-function modification^21,22^ to remove the presence of lesion during process and avoid bias. Masking the lesions from the individual images removed the potential misclassification of tissue type and distortion of normalization during the affine transformation and nonlinear adjustments. Firstly, lesion masks were manually and independently traced by 2 radiologists (W.W. and Z.Z.) in initial space according to visible damage on 3D T1-weighted images, also guided by T2-weighted FLAIR image (see Supplementary e1). Secondly, a sample-specific brain template was developed. The lesion maps identified the locations of space-OL to mask out these regions on the individual 3D T1 weighted images and normalized the lesion-masked volumes of all participants to the standard brain template of the Montreal Neurological Institute using a 12-parameter affine transformation with nonlinear adjustments with 7 × 8 × 7 basis functions. All individual normalized 3D T1 weighted anatomical images and all lesion-mask images were averaged, respectively, to yield sample-specific brain templates. Thirdly, 3D T1 weighted images were segmented using sample-specific templates. The information of spatial-transformation during this step was applied to 3D T1 weighted images for renormalization. Fourthly, voxel-based morphometry (VBM) analysis was performed using VBM8 (http://dbm.neuro.uni-jena.de/vbm) implemented in SPM8 (http://www.fil.ion.ucl.ac.uk/spm). The normalized images of each subject were again normalized into standard Montreal Neurological Institute (MNI) space with a 12-parameter affine-only nonlinear transformation, and resampled to 1.5 × 1.5 × 1.5 mm^3^. All images were segmented into 3 tissue classes representing gray matter, white matter, and cerebrospinal fluid. The resultant tissue images were further smoothed with an 8 mm full width at half maximum isotropic Gaussian kernel for subsequent morphological analyses.

#### Processing of Structural Imaging Data

For functional data, the first 5 images were removed to ensure steady-state longitudinal magnetization. Slice-timing and realignment in SPM8 were performed. Translation or rotation parameters in any given data set did not exceed ±1.5 mm or ±1.5°. Then, the functional images were coregistered to the individual 3D T1 images. The information of spatial-transformation during the third step in structural data processing was also applied to for data normalization with resample of 3 × 3 × 3 mm^3^ voxel size. After spatial smoothing (FWHM = 8 mm), the images were temporally filtered (0.01–0.08 Hz) to remove the effects of very low-frequency drift and high-frequency noises. Then, ALFF calculation was carried out using REST software (http://resting-fmri.sourceforge.net). At each voxel, the time series was transformed to the frequency domain by using fast Fourier transform. The power spectrum was then computed and square root-transformed at each voxel. The averaged square root of activity in the low-frequency band (0.01–0.08 Hz) was taken as the ALFF. The ALFF value of each voxel was standardized by dividing the full-brain mean ALFF values. Moreover, index of fractional ALFF was additionally observed by dividing the ALFF in a given frequency band to the ALFF over the entire frequency range.^23^

### Statistical Analysis

#### Group Comparisons

To compare imaging parameters among patient groups and HCs, voxel-wise 1-way analysis of variance (ANOVA) were performed using SPM8 for gray matter volume (GMV) and ALFF, respectively. Moreover, for comparison of ALFF, considering the potential effect of morphometric alteration on functional data, we additionally performed 1-way ANOVA analysis with voxel-wise regression of GMV. The outcome was superimposed on the MNI152 T1 template.

For ALFF maps, the results were estimated with a corrected threshold of *P* < 0.05, AlphaSim correction (http://afni.nih.gov/afni/docpdf/AlphaSim.pdf) (combined height threshold of *P* < 0.01 and a minimum cluster size of 26 voxels). Moreover, the results of VBM were estimated with a corrected threshold of *P* < 0.01 (combined height threshold of *P* < 0.01 and a minimum cluster size of 955 voxels). The OL in temporal lobe were covered in the process; therefore, we only observed regions outside the ipsilateral temporal lobe in the present study.

#### Correlation Analyses

Clinical variables (including epilepsy duration and seizure frequency) and neuropsychological score (including verbal intelligence quotient and performance intelligence quotient) were correlated with imaging parameters in patient groups.

For clinical variables, voxel-wise correlation analyses were performed with imaging parameters in each patient group. For neuropsychological test, due to limited number of cases, correlation was performed with imaging parameters in group of mTLE-HS and in group combining mTLE-HS and mTLE-OL. The results were thresholded at *P* < 0.05, with Alphsim correction (combined height threshold of *P* < 0.01 and a minimum cluster size of 26 voxels). In correlation analyses of ALFF, GMV was voxel-wisely regressed.

Moreover, considering the possible effect of lesion size on imaging alterations, we employed an index of damage score^24,25^ for depicting lesion size, and investigated its relationship with imaging presentations in patient groups (Supplementary e4). Damage score was identified by the number of voxels within the lesion mask in each patient.

## RESULTS

### Clinical Data

For clinical variables, patients with mTLE-HS had more frequent IPI than mTLE-OL (*t* = 7.937, *P* = 0.005, Chi-square test). No difference was found in epilepsy durations (*t* = −0.177, *P* = 0.860, 2-sample *t* test), history of secondary generalized seizures (*t* = 0.417, *P* = 0.519, Chi-square test, response to antiepileptic drug (*t* = 0.617, *P* = 0.432, Chi-square test), and surgical outcomes (*t* = 1.678, *P* = 0.195, Chi-square test) between patient groups. For neuropsychological tests, mTLE-HS had significant lower scores in PIQ (*P* = 0.047), and mild lower scores than mTLE-OL (*P* = 0.051), no difference was found in VIQ (*P* = 0.286, Mann–Whiney *U* test).

### Morphometric Alteration of GMV in mTLEs

**FIGURE 1:**
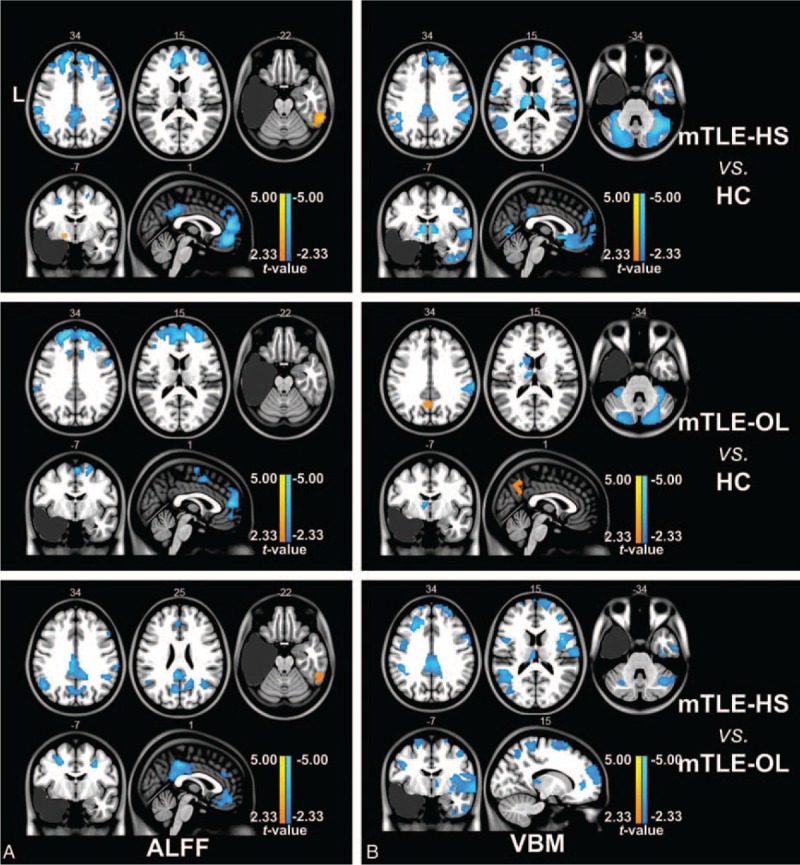
Two-sample *t* tests under 1-way ANOVA of imaging parameters between groups. (A) Group comparison of ALFF between mTLE patients and healthy controls using 2-sample *t* test (*P* < 0.01, AlphaSim correction). Both mTLE-HS and mTLE-OL patients showed decreased ALFF (labeled in cold color) in bilateral anterior cingulate cortex and frontal gyrus compared with HC group. However, mTLE-HS showed decreased ALFF in bilateral frontal cortex, inferior parietal gyrus, and posterior cingulate cortex areas compared with mTLE-OL. (B) Group comparison of GMV between mTLE patients and healthy controls using 2-sample *t* test (*P* < 0.01, AlphaSim correction). MTLE-HS and mTLE-OL showed different distribution patterns of abnormalities of GMV compared to HC. In comparison with the mTLE-OL patients, decreased GMV was distributed in contralateral insula, mesial prefrontal lobe, bilateral cingulate cortex, and precuneus in mTLE-HS. Abbreviations: ALFF = amplitude of low-frequency fluctuation, HC = human controls, HS = hippocampalsclerosis, L = left, mTLE = mesial temporal lobe epilepsy, OL = other lesions, VBM = voxel-based morphometry.

The lesion sizes in patients with mTLE-HS were smaller than those with mTLE-OL (damage scores in mTLE-HS/mTLE-OL: 867 ± 285/1390 + 1214; *t* = 2.28, *P* < 0.05). Compared with the HC group, both the patient groups showed decrease of GMV in the cerebellum and bilateral thalamus (labeled in cold color). The patients with mTLE-HS showed more wide GMV decrease in the contralateral temporal lobe, frontal lobe, thalamus, insular, and cingulate cortex. In the comparison between the patient groups, mTLE-HS showed more severe decrease of GMV in the mesial prefrontal lobe, cingulate cortex, precuneus, bilateral thalamus, and contralateral insula (Figure 1, Table 2 and Supplementary e2).

**TABLE 2:**
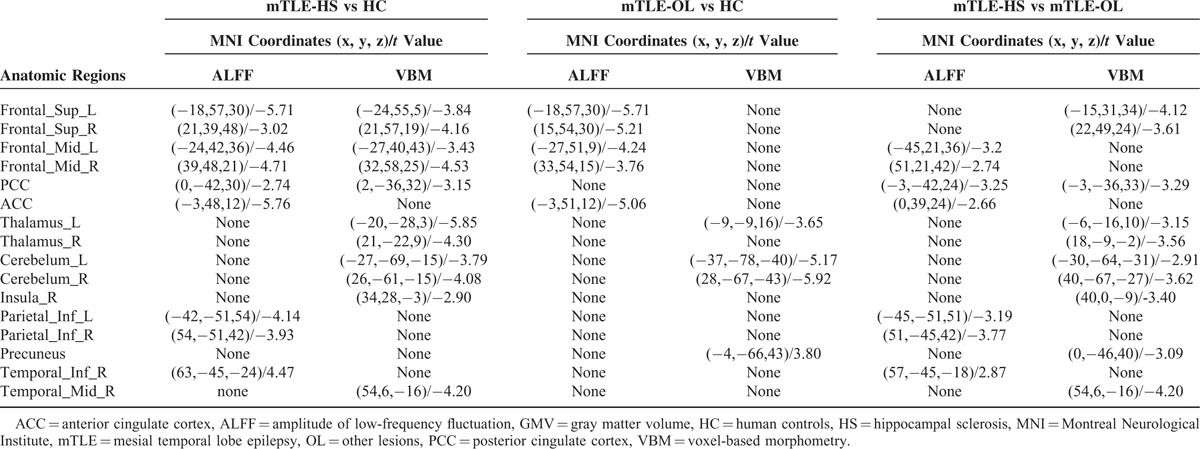
Regions of Altered ALFF and GMV in mTLE Patients

### Functional Alterations of Resting-State Brain Activity in mTLEs

Comparing with the HCs, both patient groups of mTLE-HS and mTLE-OL showed wide ALFF differences (*P* < 0.01, cluster size > 26). These 2 patients groups both showed decreased ALFF value (labeled in cold color) in the bilateral anterior cingulate coretex, middle and superior frontal gyrus, while the mTLE-HS group showed increased ALFF value (labeled in warm color) in the contralateral mesial temporal lobe. Specifically, the mTLE-HS group showed decreased ALFF in the bilateral inferior parietal gyrus and posterior cingulate cortex. Comparison between 2 patient groups showed more decrease of ALFF in bilateral frontal cortex, inferior parietal gyrus, and posterior cingulate cortex areas in mTLE-HS (Figure 1, Table 2). The result of fractional ALFF demonstrated a similar pattern with that of ALFF (Supplementary e3). In addition, we presented comparison results without GMV regression in the supplementary materials (Supplementary e4).

### Correlation Analyses

**FIGURE 2:**
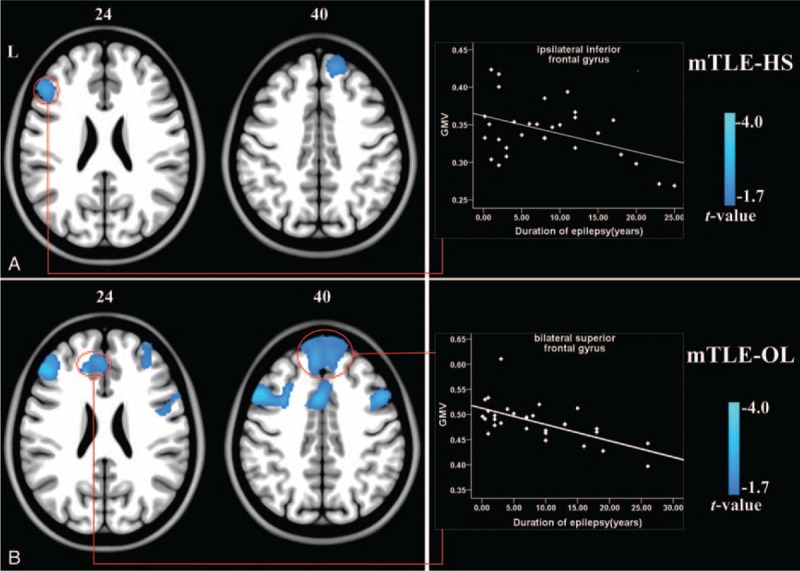
Correlation analyses between epilepsy duration and GMV of mTLE patients. (A) The frontal lobe areas in mTLE-HS patients displayed negative correlation between GMV and epilepsy durations. (B) The bilateral superior frontal gyrus in mTLE-OL patients showed negative correlation between GMV and durations. There results were thresholded at *P* < 0.05 (Alphsim correction). The right scatter plot gave an example of correlation between epilepsy duration and GMV values within the cluster marked by circle. Abbreviations: HS = hippocampal sclerosis, L = left, mTLE = mesial temporal lobe epilepsy, OL = other lesions.

Negative correlation between epilepsy duration and GMV was found in the ipsilateral inferior frontal gyrus in the mTLE-HS, and bilateral superior frontal gyrus in the mTLE-OL (Figure 2 and Supplementary e6). No correlation was found between epilepsy duration and ALFF in both patient groups. In addition, neither correlation was found between seizure frequencies and imaging parameters.

**FIGURE 3:**
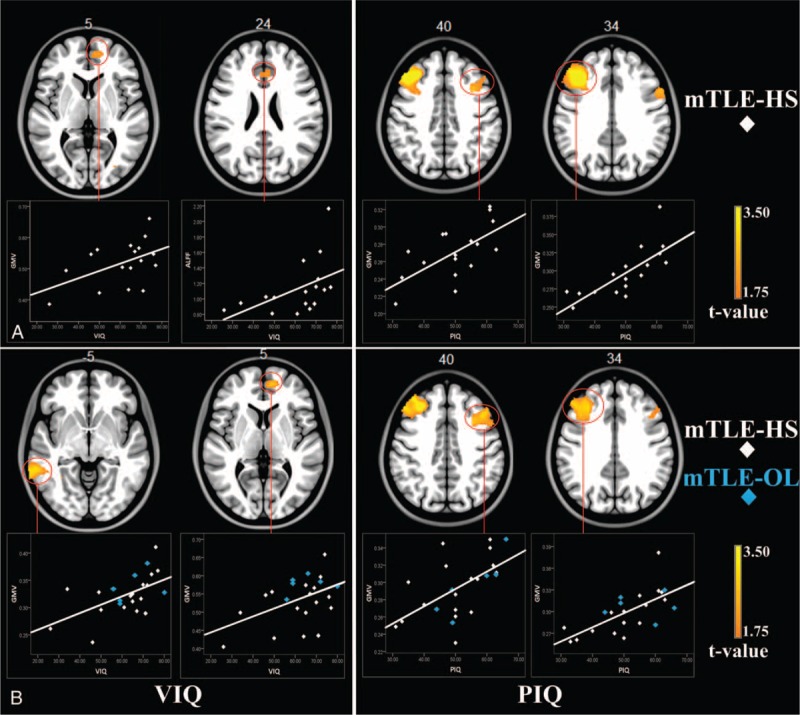
Correlation analyses between imaging parameters and neuropsychological scores. (A) The regions of bilateral anterior cingulate and mesial prefrontal lobe in mTLE-HS patients showed positive correlation between imaging parameters and VIQ. Moreover, GMV in bilateral middle frontal gyrus were positively correlated with PIQ. (B) The regions of ipsilateral superior temporal gyrus (Brodmann 22) and bilateral mesial prefrontal lobe in patients combining mTLE-HS and mTLE-OL showed positive correlation between GMV and VIQ. In addition, positive correlation between GMV and PIQ was found in the bilateral middle frontal gyrus. The scatter plot gave an example of correlation between epilepsy duration and GMV values within the cluster marked by circle. Abbreviations: HS = hippocampal sclerosis, L = left, mTLE = mesial temporal lobe epilepsy, OL = other lesions, PIQ = performance intelligence quotient, VIQ = verbal intelligence quotient.

In patients with mTLE-HS, positive correlation between GMV and VIQ was found in the bilateral anterior cingulate and mesial prefrontal lobe, moreover, positive correlation between ALFF and VIQ was found in the anterior cingulate cortex. In the patients combining mTLE-HS with mTLE-OL, positive correlation between GMV and PIQ was found in the bilateral middle frontal gyrus (Figure 3, Supplementary e7). Moreover, for the correlation between damage scores and imaging parameters, the correlation results did not spatially overlap with group comparison results (see Supplementary e5).

## DISCUSSION

This study combined ALFF-based fMRI and VBM-based morphometric MRI techniques to investigate the alterations of functional and structural organizations in mTLE with HS and OL. Both mTLE-HS and mTLE-OL showed decreased resting-state brain activity and GMV relative to healthy controls mainly in the frontal cortex. Importantly, despite with smaller lesion size of the pathological focus, mTLE-HS presented more severe decreases of ALFF and GMV in the extra-temporal regions. The GMV in the frontal cortex was negatively correlated with epilepsy duration. This study provided imaging evidence to demonstrated that mTLE-HS is different from mTLE-OL in the wide extra-temporal regions other than pathological focus.

We used 2 parameters for describing the regional properties of brain structure and function, to investigate the imaging alterations in mTLE with different pathological forms. In line with previous studies of ours^15^ and others,^26,27^ we found decreased ALFF in the anterior and posterior cingulate cortex, and medial prefrontal cortex in mTLE-HS, which was explained as high-level cognitive impairments, such as brain default function and executive function. We also found that the VIQ scores were positively correlated with ALFF in the anterior cingulate, indicating that decreased ALFF might be related with deficit of cognitive functions. Moreover, decreased GMV was found in the frontal lobe, thalamus, cingulate cortex, and cerebellum. This result was consistent with previous VBM studies^28^ on mTLE. Negative correlation between GMV and epilepsy duration implied progressive structural damages in the extra-temporal structures caused by epileptic activity. Importantly, we for the first time displayed functional and structural imaging alterations in mTLE-OL. The patients also showed decreased ALFF in the bilateral middle and superior frontal gyrus, and anterior cingulate cortex, decreased GMV in the ipsilateral caudate nucleus, putamen, thalamus, and cerebellum in comparison with healthy controls. Decreased ALFF in the frontal cortex and decreased GMV in the thalamus and cerebellum were consistent with imaging alterations in mTLE-HS. Similar to the explanation for mTLE-HS, we suggest that the imaging results reflect functional and structural damages associated with cognitive deficits and disease progression.

Comparisons between patient groups showed different imaging presentations in mTLE-HS and mTLE-OL. Despite with smaller lesion size of the pathological focus, mTLE-HS showed more extensive decreases of ALFF and GMV in extra-temporal regions relative to mTLE-OL. These findings indicated that mTLE-HS might have more severe functional and structural damages. This work used strict criteria for patient inclusion of mTLE-HS, in order to match the lateralization, genders and age of mTLE-OL, and there was no difference in disease severity (epilepsy duration, seizure frequency, and history of generalized seizures) between patient groups. Thus, the imaging differences might not be caused by the above factors. Moreover, smaller lesion size and more extra-temporal damage in the mTLE-HS group, and negative result of correlation between damage scores and imaging parameters both implied that extra-temporal imaging differences between patient groups might not be correlated with extent of lesions.

We found that mTLE-HS showed lower neuropsychological score in PIQ than mTLE-OL, which implied that mTLE-HS have more severe deficit of higher-cognitive functions.^2,7,8^ Correlation between imaging parameters and cognitive scores within the frontal cortex further suggested that more decreases in ALFF and GMV might be related with severe cognitive deficits in executive function. Thus the present study provided solid imaging evidence to support the assumption that mTLE-HS has more severe brain damage. In pathological basis, HS is loss of neuronal and hyperplasia of glia cell,^29^ loss of hippocampal connections can lead to remote deafferentation^30,31^ and thereby neuronal damage, particularly since structures directly connected to the hippocampus.^32^ Whereas, for foreign-tissue lesions, such as benign brain tumor, exert compressing effect on brain tissue,^33^ may cause less functional damage on neuronal cells, and lead to milder functional structural alterations in the extra-temporal regions. The present findings implied that mTLE-HS should received earlier intervention for effective seizure control because more severe brain damage.

There remains controversy about the difference of surgical outcomes between mTLE-HS and mTLE-OL. A few of studies^34–36^ and the present work with limited available data displayed that no difference of surgical outcomes existed between these 2 patient groups, the other studies^8,11,37^ implied that mTLE-HS might have more satisfactory outcome than mTLE-OL. We suspected that different brain organizations might be correlated with surgical outcomes in mTLE.^38–42^

A few of limitations in this study should be noted. First, considering the potential influences of clinical and demographic variables (such as disease severity, lateralization, age, and gender) on brain organization, we selected patients with mTLE-HS to match these variables of mTLE-OL. Although patient selection was limited to a cohort of consecutive data within a period of time, the operations might cause the credibility in clinical presentation, such as surgical outcomes. Second, only a portion of patients underwent neuropsychological test and surgical treatment. Small number of cases would lower reliability of statistical power. Third, although we estimated the relationship between damage scores of lesions and imaging presentations in extra-temporal regions, the potential influence of lesion sizes on extra-temporal damages might not be excluded. Fourth, mTLE-OL in the present study was defined to involve a cohort of heterogeneous pathological substrates other than HS. Study on homogenous subtype would be benefit to more clearly clarifying the specific mechanism of mTLE with different pathological substrates. Finally, potential confounding of partial volume effects and susceptibility artifact of the functional data should be noted.

## CONCLUSIONS

By employing ALFF-based functional MRI and VBM-based morphometric MRI techniques, we for the first time revealed different alteration patterns of functional and structural organization in mTLEs with HS and OL. Despite with smaller lesion size of the pathological focus, mTLE-HS presents more severe functional and structural brain damages in the frontal cortex than mTLE-OL. The results had implication that mTLE-HS should received earlier intervention for effective seizure control because more severe brain damage, and also provided imaging evidence to support the proposal that mTLE-HS is a special epilepsy syndrome.

## Supplementary Material

Supplemental Digital Content
