# Supplementary material for: More Severe Extratemporal Damages in Mesial Temporal Lobe Epilepsy With Hippocampal Sclerosis Than That With Other Lesions: A Multimodality MRI Study
Source: Medicine (Baltimore). 2016 Mar 11;95(10):e3020. doi: 10.1097/MD.0000000000003020 (PMC4998901; doi:10.1097/MD.0000000000003020)
Supplement: Supplemental Digital Content [file medi-95-e03020-s001.pdf]

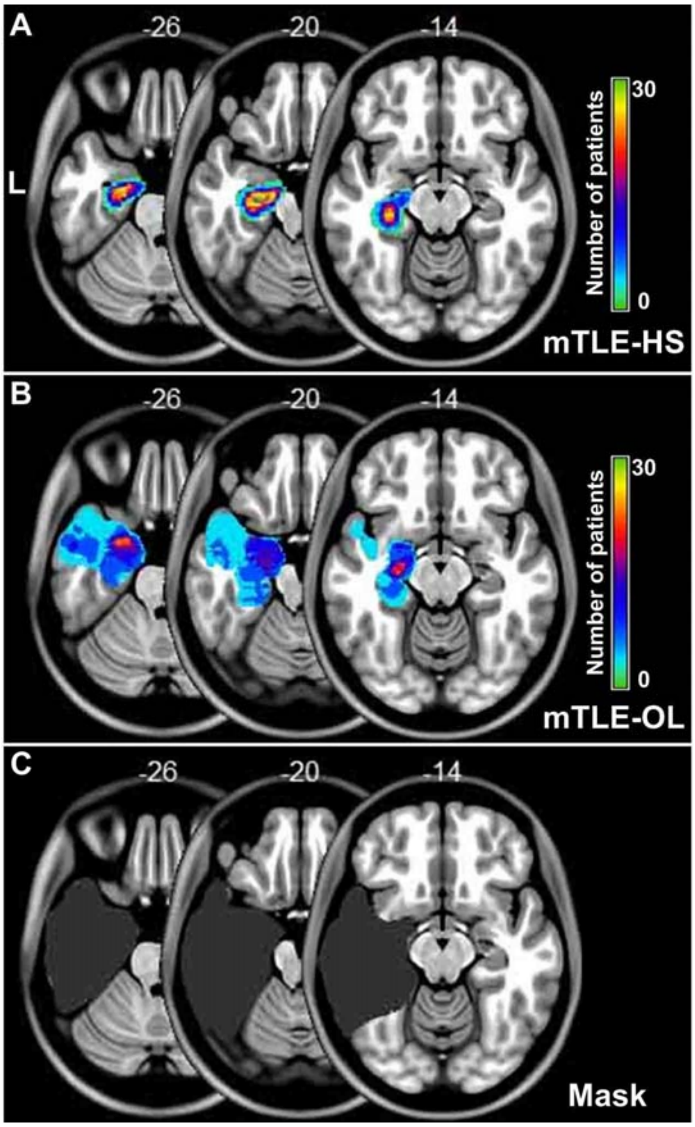

Supplementary e1 Overlaps of lesions and mask of mTLEs.

Overlays of lesions of all patients with mTLE-HS (**A**) and mTLE-OL (**B**). Colours represent number of patients with a lesion to a specific voxel. Warmer areas indicate areas of greater lesion overlap. Colour range runs from 1 (the lowest value in the image) to 30 (the highest value in the image). **C:** Mask for cost-function normalization processing, which covers all temporal lobe of lesioned side.

Abbreviations: L= left, mTLE= mesial temporal lobe epilepsy, HS= hippocampal sclerosis, OL= other lesions.

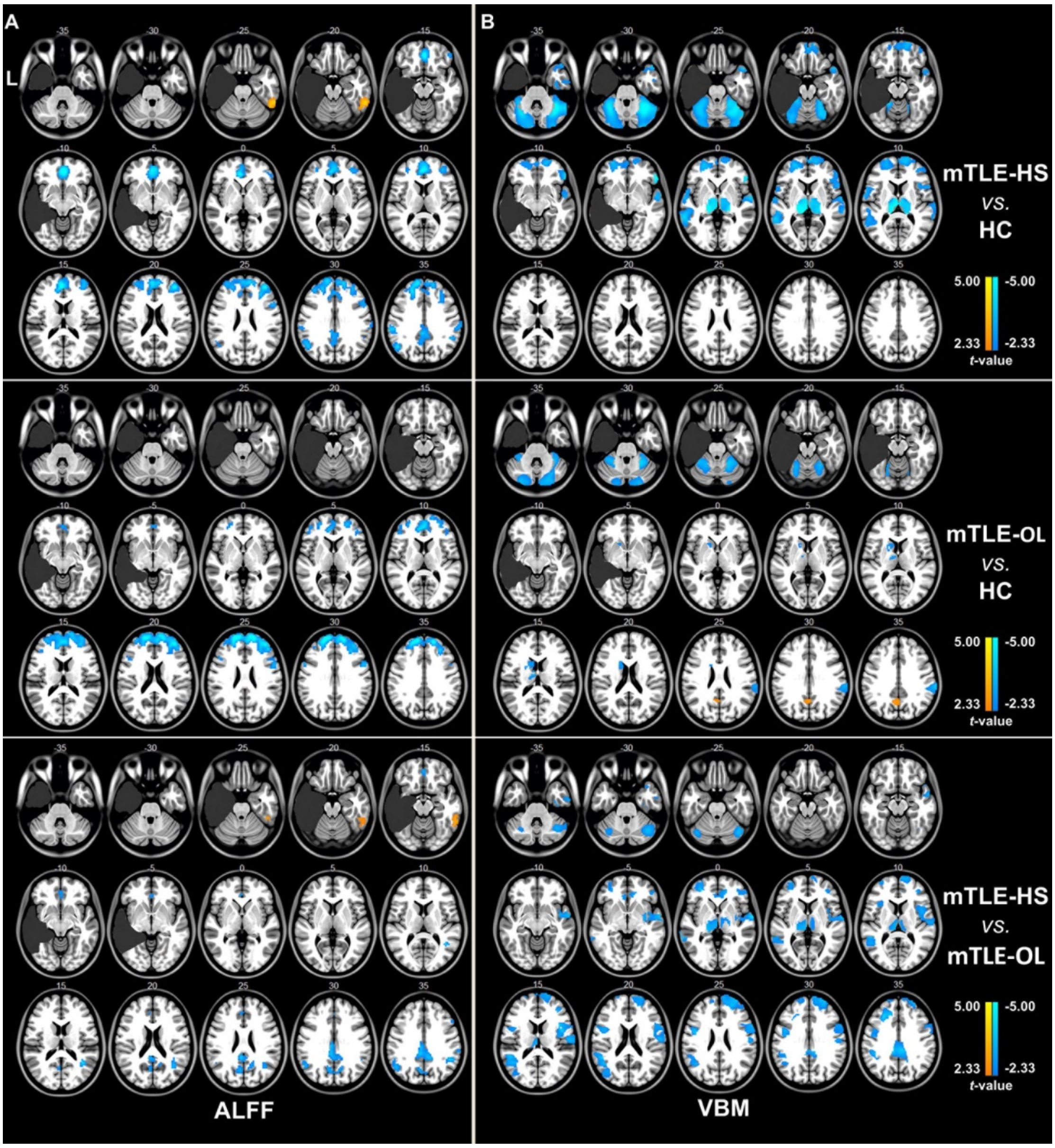

Supplementary e2. Two-sample t-tests under one way ANOVA of imaging parameters between groups.

The figure was shown with consistent slices covering the entire brain.

Abbreviations: L= left, mTLE= mesial temporal lobe epilepsy, HS= hippocampalsclerosis, HC= human controls, OL= other lesions, ALFF= amplitude of low frequency fluctuation, VBM= voxel-based morphometry.

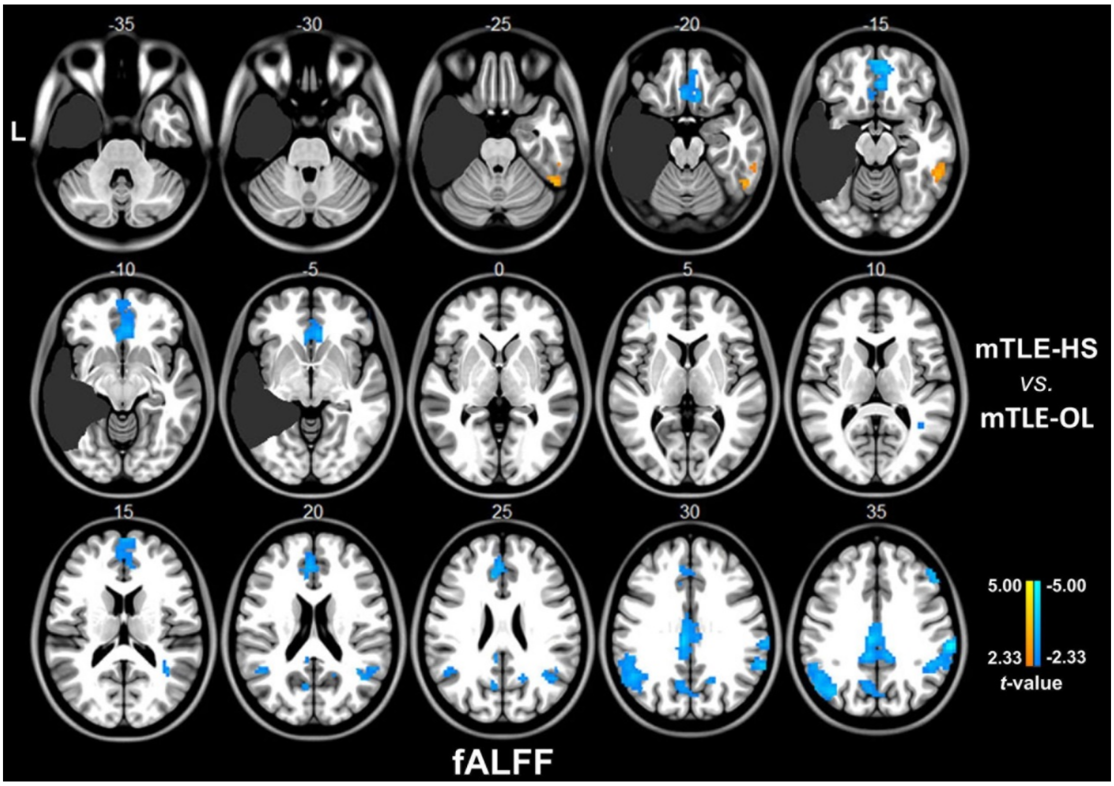

Supplementary e3. Two-sample t-tests under one way ANOVA of fractional ALFF between patients with mTLE-HS and mTLE-OL.

The results demonstrated a similar pattern with that of ALFF (Supplementary material figure 2).

Abbreviations: L= left, mTLE= mesial temporal lobe epilepsy, HS= hippocampalsclerosis, OL= other lesions, fALFF= frational amplitude of low frequency fluctuation.

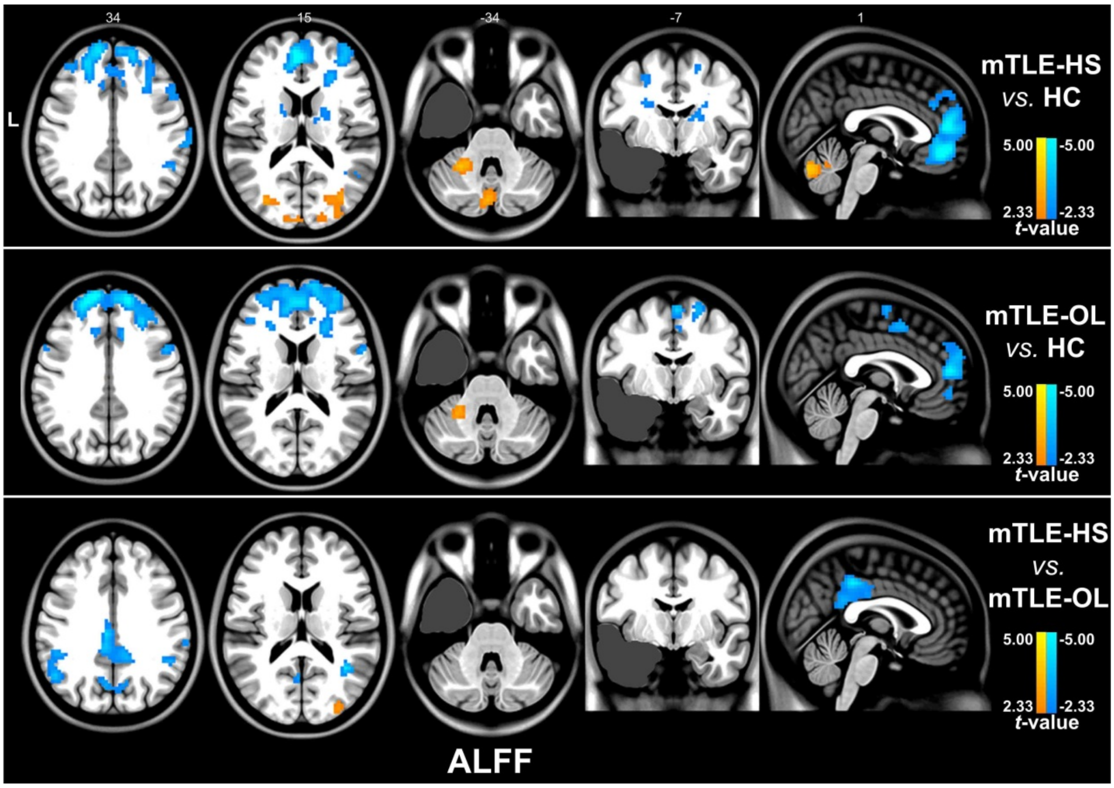

Supplementary e4. Comparisons of ALFF without GMV regression.

The mTLE-HS and mTLE-OL both showed increased mALFF (labeled in warm color) in bilateral cerebellum compared to HC, whereas decreased ALFF(labeled in cold color) was found in anterior cingulate cortex, bilateral middle and superior frontal gyrus. In addition, mTLE-HS showed decreased ALFF in inferior parietal gyrus and posterior cingulate cortex areas compared to mTLE-OL.

Abbreviations: mTLE= mesial temporal lobe epilepsy, HS= hippocampal sclerosis, OL= other lesions, HC= human controls, L= left, ALFF= amplitude of low frequency fluctuation.

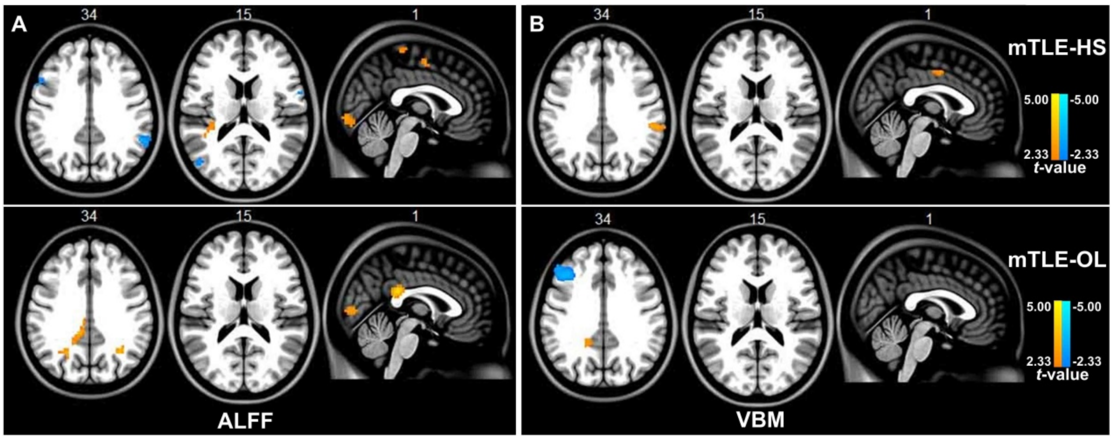

Supplementary e5. Correlation analyses between damage score and imaging presentations of mTLE patients.

Damage score was identified by the number of voxels of lesion in each patient.

A: Correlation analyse between damage score and ALFF of mTLE patients. The regions of contralateral mesial temporal lobe, bilateral frontal lobe and parietal lobe in mTLE-OL patients showed positive correlation between damage score and ALFF (labeled in warm color), moreover, bilateral parietal lobe in mTLE-HS patients showed positive correlation between damage score and ALFF. **B:** Correlation analyse between damage score and VBM of mTLE patients. Positive correlation between damage score and VBM were found in the contralateral frontal lobe and cerebellum, bilateral thalamus and parietal lobe in mTLE-HS patients. In addition, no correlation was found between damage score and VBM in mTLE-OL patients.

Abbreviations: mTLE= mesial temporal lobe epilepsy, HS= hippocampal sclerosis, OL= other lesions, ALFF= amplitude of low frequency fluctuation, VBM= voxel-based morphometry.

Supplementary e6 Regions of gray matter volume negatively correlated with durations of epilepsy in the patients with mesial temporal lobe epilepsy with hippocampal sclerosis and the patients with mesial temporal lobe epilepsy with other lesions

Anatomic region	mTLE-HS	mTLE-OL
	MNI coordinates(x,y,z)/*t* value	MNI coordinates(x,y,z)/*t* value
Medial frontal gyrus	(16,50,43)/-2.71	(-12,36,34)/-3.93
Lateral frontal gyrus	(-48,28,24)/-2.99	(-49,25,33)/-3.27

Abbreviations: mTLE= mesial temporal lobe epilepsy, HS= hippocampal sclerosis, OL= other lesions, MNI= Montreal Neurological Institute.

Supplementary e7 Regions of imaging parameters positively correlated with neuropsychological scores in the patients with mesial temporal lobe epilepsy with hippocampal sclerosis and the patients with mesial temporal lobe epilepsy with other lesions

Patients	VIQ		PIQ	
	Anatomic region	MNI coordinates(x,y,z)/t value	Anatomic region	MNI coordinates(x,y,z)/t value
mTLE-HS	anterior cingulate	(14,52,2)/2.35	ipsilateral middle frontal gyrus	(-41,25,34)/4.82
mTLE-HS	mesial prefrontal lobe	(6,27,27)/2.38	contralateral middle frontal gyrus	(33,15,40)/2.62
mTLE-HS and mTLE-OL	ipsilateral superior temporal gyrus	(-66,-46,-5)/3.02	ipsilateral middle frontal gyrus	(-28,34,45)/5.78
mTLE-HS and mTLE-OL	mesial prefrontal lobe	(12,52,3)/2.73	contralateral middle frontal gyrus	(44,25,45)/3.73

Abbreviations: VIQ= verbal intelligence quotient, PIQ= performance intelligence quotient, MNI= Montreal Neurological Institute, mTLE= mesial temporal lobe epilepsy, HS= hippocampal sclerosis, OL= other lesions.
